# Angiopoietin-2: A New Therapeutic Target for Preventing Lung Dysfunction in Sepsis?

**DOI:** 10.1371/journal.pmed.0030097

**Published:** 2006-01-24

Every year, more than 200,000 Americans die from sepsis, a severe illness caused by bacterial infection of the bloodstream. In the United States, 2%–3% of all hospital admissions are for sepsis, which has a mortality rate of about 30%. In sepsis, pathogens that have entered the bloodstream through a wound or from an infected organ induce a systemic inflammatory response, which can cause circulatory system damage, multiple organ dysfunction, and death, particularly in people with other medical conditions.

Treatment for sepsis includes antibiotics to clear the initiating bacteria and to provide medical support for damaged organs—mechanical ventilation for lung dysfunction, for example. However, the prognosis for patients would be much better if organ dysfunction could be prevented, so researchers, including Samir Parikh, Vikas Sukhatme, and their colleagues, are investigating how organs become damaged during sepsis. Parikh's team now report results suggesting that a protein called angiopoietin-2 may play a pivotal role in acute respiratory distress syndrome (ARDS), a condition that affects about 40% of patients with sepsis and worsens their prognosis.

ARDS starts with protein-rich fluid leaking out of the blood capillaries of the lung into the alveoli, the terminal airspaces where gas exchange normally occurs. The presence of this fluid in the alveoli impairs gas exchange, and prevents the lungs from expanding and contracting normally during breathing. But what causes fluid to leak out of the vasculature? Capillaries are normally lined with endothelial cells linked together with adhesive junctions to form an impermeable barrier. The development of this barrier is controlled in part by two peptides called angiopoietin-1 (Ang-1) and angoipoietin-2 (Ang-2). Both peptides bind as ligands to a signaling receptor called Tie-2. Ang-1 functions as an agonist; its binding activates the Tie-2 receptor. In contrast, Ang-2 is an antagonist, whose binding blocks signal transduction. Animal experiments have shown that the two ligands have opposite functions in vascular development: Ang-1 promotes the stabilization of nascent blood vessels; Ang-2 disrupts this action. These findings, the fact that Tie-2 expression may be abnormal in bronchopulmonary dysplasia (a developmental lung disorder), and the high level of Tie-2 expression in lungs prompted Parikh's team to ask if the Tie-2 signaling pathway is affected in sepsis and, specifically, if excess Ang-2 levels might promote the vascular leakage that underlies ARDS.

The researchers measured serum Ang-2 concentrations in 22 patients admitted to the hospital with sepsis, as well as in 29 control individuals hospitalized with other conditions. Admission levels of Ang-2 were higher in patients with septic shock (characterized by low blood pressure) or multiorgan failure than in control patients or in patients with sepsis alone. Over time, Ang-2 levels mirrored the clinical course of illness in individual patients, increasing as they became more sick and declining as they recovered. Finally, Ang-2 levels correlated with impaired lung function. These results are consistent with the idea that increased circulating Ang-2 might contribute to vascular leakage in sepsis.

To test their hypothesis further, the researchers turned to thin sheets (monolayers) of cultured endothelial cells. The addition of patient serum containing high levels of Ang-2 (but not serum containing low levels) to these monolayers caused changes in the actomyosin cytoskeleton (a network of proteins that forms the cellular skeleton). These structural changes, which resulted in the endothelial cells contracting and gaps forming between them, could be replicated by the addition of Ang-2 alone, and were accompanied by increased permeability of the endothelial monolayer. Adding Ang-1 reversed the effects of Ang-2 on the endothelial cells. Further experiments indicated that Ang-2 activated a protein called Rho-kinase and the subsequent addition of phosphate groups to myosin light chain, a protein involved in cell contraction. In the last set of experiments, the researchers showed that systemic administration of Ang-2 to healthy mice increased vascular permeability and caused fluid extravasation in their lungs, changes reminiscent of early ARDS.

These results support a role of Ang-2 in sepsis-associated ARDS, and suggest new targets for the treatment of sepsis. Up until now, the emphasis has been on damping down the systemic immune response, but drugs that do this often have unwanted side effects. Targeting Tie-2 signaling might be a cleaner way to deal with the leaky vasculature that causes ARDS and other aspects of sepsis. In addition, suggest Parikh and colleagues, serial Ang-2 measurements might identify which patients at risk of developing ARDS would benefit most from therapy aimed at preventing vascular leakage. But, they acknowledge, further experiments are needed to prove conclusively that Ang-2 plays a causal role in human ARDS, to clarify why the lungs are especially susceptible to systemic excess levels of Ang-2, and to test whether interfering with Ang-2 function can yield therapeutic benefits.

## 

**Figure:**
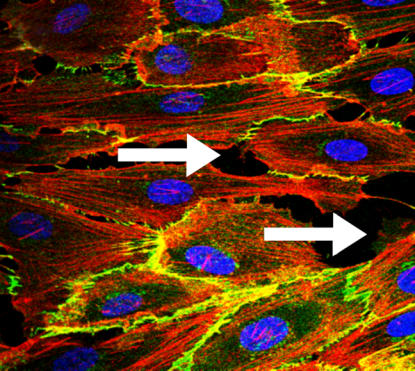
Ang-2 causes intercellular gaps in cultured endothelial cells

